# Phytochemical profiling, antimicrobial, and antioxidant activities of hydroethanolic extracts of prickly pear (*Opuntia ficus indica*) fruit and pulp

**DOI:** 10.1002/fsn3.3226

**Published:** 2023-01-22

**Authors:** Khansa Iftikhar, Farzana Siddique, Kashif Ameer, Muhammad Arshad, Sadia Kharal, Isam A. Mohamed Ahmed, Zarina Yasmin, Nida Aziz

**Affiliations:** ^1^ Institute of Food Science and Nutrition, University of Sargodha Sargodha Pakistan; ^2^ Department of Zoology University of Sargodha Sargodha Punjab Pakistan; ^3^ Department of Food Science and Technology, Faculty of Agriculture University of Khartoum Shambat Sudan; ^4^ Department of Food Science and Nutrition College of Food and Agricultural Sciences, King Saud University Riyadh Saudi Arabia; ^5^ Post Harvest Research Centre Ayub Agricultural Research Institute Faisalabad Pakistan; ^6^ Department of Zoology University of Punjab Lahore Pakistan

**Keywords:** antioxidants, cladode, flavonoids, *Opuntia*, polyphenols

## Abstract

Phenolic compounds in prickly pear [*Opuntia ficus indica* (L.) Mill.] are known to contribute to the antioxidant and antimicrobial activities of the prickly pear. The present study aimed to evaluate the antioxidants and in vitro antimicrobial potential in the hydroethanolic extracts of different parts (fruit, cladode, and pulp) of prickly pear. Different polyphenolic compounds were analyzed by using high‐performance liquid chromatography. The results indicated that cladode possessed a higher quantity of phenolics compared with that observed in fruit and pulp. The most important phenolic compound in high quantity was gallic acid (66.19 μg/g) in cladode. The 100% aqueous extract of cladode exhibited the highest antioxidant (92%) and antimicrobial activities against *Salmonella typhi* (3.40 mg/ml), *Helicobacter pylori* (1.37 mg/ml), *Escherichia coli* (1.41 mg/ml), and *Staphylococcus aureus* (1.41 mg/ml). Principal component analysis (PCA) indicated that antioxidant activity and minimum inhibitory concentration (MIC) responses had a significant negative correlation with each other. Overall, the current results provided basic data for choosing prickly pear cladode with high antioxidant capacity for the development and consumption of antioxidant‐based alternative medicines and value addition of formulated foods.

## Graphical abstract

Prickly pear fruit and pulp contained high total phenolic, flavonoid, and carotenoid contents with strong antioxidant potential. The prickly pear exhibited antimicrobial potential against various microbial strains. TPC, TFC, and TC exhibited a positive correlation with antioxidant activity, whereas MIC exhibited a negative correlation with that DPPH activity.

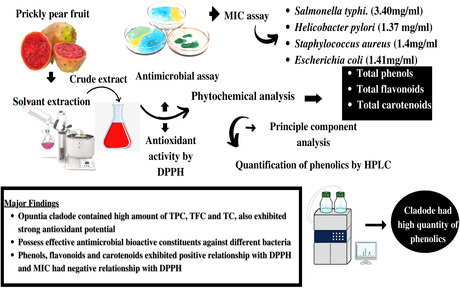

## 1 INTRODUCTION

The increasing demands of water and feed resources across the world's dry areas require alternative sources of animal feed, specifically crops with better water‐use efficiency (Ameer, Bae, Jo, Lee, et al., 2017). One alternative with the potential for widespread contribution toward reducing the impact of reduced feed and water availability is the cactus pear. *Cactaceae Juss* (1789) is a highly diversified family of xerophytes that are dominant in the arid and semi‐arid environments of the Americas, which is its center of origin and diversification (Hernández‐Hernández et al., 2014). Cacti comprise approximately 1400–1800 described species in the world (Guerrero et al., 2019), and Mexico is the country with the greatest diversity, with 52 genera and 850 species, of which an estimated 84% are endemic (Guerrero et al., 2019). Nearly, 31% of cacti are globally threatened (Goettsch et al., 2015) due to changes in land use, the introduction of exotic species, and uncontrolled harvesting of these plants for use as food, raw materials, and other purposes as well as influences by climate change. Some of them are listed by the International Union for the Conservation of Nature (IUCN) under various threat categories which necessitate conservation efforts. *Opuntioideae* is the richest genus within the *Cactaceae* of nearly 200 described species (Huo et al., 2022; Majure et al., 2012; Majure & Puente, 2014). *Opuntia* species are highly distributed throughout arid and semi‐arid environments and are well‐adapted to drought‐stressed conditions (Aliscioni et al., 2021; Majure et al., 2012). The genus *Opuntia* is endemic to the Americas and distributed from regions of Canada to Argentina (Majure & Puente, 2014), showing a high number of regional endemic species in Mexico (González‐Elizondo et al., 2017). Prickly pear fruit has an aqueous pulp and contains 87.5% of water. It has a low energy value of 170 kJ/100 g. Its carbohydrate content, mainly glucose and fructose, provides 94% of the energy value. It exhibits a low titratable acidity of 1.83 g citric acid/kg than oranges, pineapple, and bananas (Garcia‐Amezquita et al., 2018).

In recent years, much attention has been devoted to natural antioxidants and their association with health benefits provision (Ameer, Chun, et al., 2017). Plants are potential sources of natural antioxidants. It produces various antioxidant compounds to counteract reactive oxygen species (ROS) in order to survive (Jiang et al., 2021). ROS, which include free radicals, such as superoxide anion radicals (O^−2^), hydroxyl radicals (–OH), and nonfree radical species, like H_2_O_2_ and single oxygen are various forms of activated oxygen (Ameer et al., 2022; Ameer, Bae, Jo, Chung, et al., 2017; Kang et al., 2022). These molecules are exacerbating factors in cellular injury and aging processes. In foods, ROS can cause lipid peroxidation, which leads to the deterioration of the foodstuffs (Li et al., 2020; Yang et al., 2021). The oxidative deterioration of lipid‐containing foods is responsible for the rancid odors and flavors during processing and storage, consequently decreasing the nutritional quality and safety of foods due to the formation of potentially toxic secondary compounds. The addition of antioxidants is a method for increasing the shelf life of foodstuffs (Ameer et al., 2017; Jiang, Lee, Ameer, & Eun, 2019; Lee et al., 2002). The antioxidant activity of prickly pear is comparable to that of red oranges and grapes (Cano et al., 2017). It exerts biological effects, which may be due to the synergistic action of betalains (tyrosine‐derived pigments), flavonoids, and other biologically active components (Stintzing et al., 2005). All parts of the cactus plant are rich in polyphenols including various flavonoids and phenolic acids. The total phenolic content (TPC) of prickly pear fruit pulp has been reported up to 218.8 mg gallic acid equivalents (mg GAE)/100 g fresh fruit in red‐skinned fruit. Prickly pear fruit is considered a rich source of flavonols. The quantification of five types of flavonoids showed that quercetin was the predominant one (58.7% ± 54.3%), followed by isorhamnetin (31.7% ± 18.8%), luteolin (11.5% ± 5.4%), and kaempferol (11.0% ± 4.8%; Fernández‐López et al., 2010). The analysis of the peels showed that isorhamnetin glycosides, especially isorhamnetin‐3‐O‐rutinoside, are the main flavonol glycosides in prickly pear peels (Moussa‐Ayoub et al., 2011). More than 20 polyphenolic compounds, including flavonoids and tannins were also detected in the prickly pear seeds.

Cactus pear contains bioactive compounds, such as quercetin, isorhamnetin, and kaempferol. These bioactive compounds have significant antimicrobial activity against different microbes including *Bacillus subtilis, Escherichia coli, Psedomonas auregnosa*, and *Klebsiella pneumonia* (Nenaah, 2013). Food‐borne bacteria resulting in gastroenteritis in humans is *Campylobacter*. The aqueous, ethanolic, and methanolic extracts of *Opuntica ficus indica* have significant antimicrobial activity against *Vibrio cholera* (Sánchez et al., 2010). Prickly pear (*O. ficus indica*) has pharmacological significance because it prevents the disorders of gut and diseases like food poisoning due to *Vibrio cholera* and *Campylobacter*. Belay et al. (2015) revealed that the ether and alcoholic extracts of Cactus pear have significant antimicrobial activity against different microbes (*S. marcescens*, *E. coli*, and *S. thermophilus*). The antimicrobial activity of cactus pear extracts was tested against different bacterial stains (*E. coli*, *Enterobacter cloacae*, *Acinetobacter baumannii*, *Proteus mirabilis*, *Citrobacter freundii*, *Klebsiella pneumoniae*, *Salmonella* spp., *Pseudomonas aeruginosa*, *Streptococcus pneumoniae*, *Enterococcus faecium*, *Staphylococcus aureus*, and *Enterococcus faecalis*) that are resistant to different drugs. The constantly increasing demand for neutraceuticals is paralleled by a more pronounced request for natural ingredients and health‐promoting foods. *Opuntia ficus indica* has gradually attained economic importance in agriculture and the wider international community through the United Nations (UN) Food and Agriculture Organization (FAO). *Opuntia* has several health‐beneficial properties so there is a need to explore possible nutraceutical compounds. The aim of the present study was to determine the minimum inhibitory concentration (MIC) against different microbes, antioxidant capacity using DPPH assay as well as the phytochemical compositions and polyphenolic contents of *Opuntia ficus indica* cladode, pulp, and fruit.

## 2 MATERIALS AND METHODS

### 2.1 Raw material collection and preparation

Red pink‐colored prickly pear fruits of the cultivar (Meyer) with cladodes were brought from Talagang district, Chakwal, Pakistan. The samples were carried to the laboratory of the Institute of Food Science and Nutrition, University of Sargodha, Sargodha. Fruits and cladodes were washed separately to remove dirty materials, air‐dried, and stored in air‐tight glass containers at 4–6°C for use in different analyses.

### 2.2 Preparation of aqueous and ethanolic extracts of cladodes

Fresh cladodes extraction was carried out according to Benayad et al. (2014) with few modifications. Cladodes (20 g) were ground and mixed with different concentrations of solvents (ethanol and water) as shown in Table 1, which were then put in a separate conical flask and these were placed in a shaking incubator (Shing Saeng Skir‐601L) at 22–25°C for 24 h. Macerated extracts were then filtered using Whatman No. 1 filter paper. Then, water and ethanol were evaporated in a rotary evaporator (Heidolph Laboratory, 4001) at 45°C and 60 rpm (Benayad et al., 2014).

**TABLE 1:** General linear model (GLM) evaluating the effect of different extracts and parts of *Opuntia ficus indica* on phenolics, flavonoids, carotenoids, and DPPH

Source of variation	df	Phenolics		Flavonoids		Carotenoids		DPPH	
		*F*	*p*	*F*	*p*	*F*	*p*	*F*	*p*
Solvent fractions	5	67.98	.00	255.91	.00	75.14	.00	7793.14	.00
Parts	2	228.69	.00	615.83	.00	681.07	.00	33294.3	.00
Treatments × parts	10	15.61	.00	104.63	.00	54.25	.00	55.85	.00

### 2.3 Preparation of extracts of fruit and pulp

Fruit and pulp extraction was performed following the protocol of Abou‐Elella and Ali (2014) with few modifications. Fruits and pulp (20 g) were ground with different concentrations of ethanol and water (Table 1) and transferred in separate conical flasks. These flasks were placed in a shaking incubator (Shing Saeng Skir‐601L) at 22–25°C for 24 h. Macerated extracts were filtered using Whatman No. 1 filter paper (Whatman Ltd.). Water and ethanol were evaporated in a rotary evaporator Laborota 4001 (Heidolph) at 45°C and 60 rpm (Abou‐Elella & Ali, 2014).

### 2.4 Quantitative phytochemical screening of cactus pear whole fruit, pulp, and cladode

Quantitative phytochemical screening was done using a spectrophotometer (UV‐1800, Shimadzu Instruments Mfg. Co., Ltd) and high‐performance liquid chromatography (HPLC; Agilent‐1260 HPLC system, Agilent Technol.).

#### 2.4.1 Total phenolic content

TPC was determined following the protocol described by AOAC method number 2017.13. For the assay, 0.25 ml of sample was taken in test tubes, mixed with 1.25 ml 10% Folin–Ciocalteu reagent, and diluted with distilled water 10 times. This solution was mixed with 1 ml of 7.5% sodium carbonate and incubated for 30 min in the dark. The absorbance was measured at 765 nm by spectrophotometer (Shimadzu Instruments). The TPC was expressed as gallic acid equivalents in milligrams (mg GAE mg)/100 g of FW.

#### 2.4.2 Total flavonoid content

TFC was determined following the method of Chougui et al. (2013) with few modifications. For the reaction, 1.5 ml of extract was taken in a test tube and incubated with 2% of 1.5 ml AlCl_3_ reagent for 30 min in darkness. The absorbance was recorded at 430 nm by the spectrophotometer. A calibration curve was prepared using quercetin as the standard. The results were expressed as mg equivalent of quercetin (mg QE)/100 g of FW (Chougui et al., 2013).

#### 2.4.3 Total carotenoids content

Chlorophyll contents (Chl a and Chl b) were determined by spectrophotometry through the reported method of Braniša et al. (2021). Total carotenoid content was quantified with few modifications. Ethanol–water in the ratio of 4:1 was employed as the extraction solvent. For the assay, 5 ml of sample was centrifuged for 5 min at 5000 rpm at 5°C in a Z383K Hermle centrifuge. The top layer of solvent had been recovered and transferred to a 25 ml volumetric flask. The spectrophotometric absorption spectra were recorded for Chl a, Chl b, and total carotenoid content at wavelengths of 663.6, 647, and 470 nm, respectively. Absorbance was measured at 450 nm by the spectrophotometer (Shimadzu Instruments). The results were expressed as the microgram equivalent of beta carotene (μg/ml). Following equations were used for the measurement of Chl a, Chl b, and total carotenoid contents.

(1) Chlorophyllaμgml=12.25A663.6—2.25A646.6,

(2) Chlorophyllbμgml=20.31A646.6—4.91A663.6,

(3) Total carotenoid contentμgml=1000A470—2.27Chla—81.4Chlb/227.

#### 2.4.4 Free radical scavenging activity by DPPH

Free radical scavenging activity was determined by using the method of Shen et al. (2010) with minor modifications according to AOAC SMPR method number 2011.011. For the assay, 750 μl of the extract was added to 1.75 ml of 0.02 g/L DPPH dissolved in ethanol. This mixture was incubated for 30 min in the darkness. The absorbance was measured at 517 nm against control by the spectrophotometer. Ascorbic acid was taken as the reference compound. The free radical scavenging activity of DPPH has been calculated by using the following formula:

$$\text{DPPH scavenging effect}\;\left(\%\text{Inhibition}\right)=\frac{A_{0}-A_{1}}{A_{0}}\times 100,$$

where *A*_0_ is the absorbance of the control and *A*_1_ is the absorbance of the extract.

### 2.5 High‐performance liquid chromatography

Flavonoids and phenolics were quantitatively analyzed by HPLC as per the reported method of AOAC, 2000 Official Method 983.15. UV visible detector (Model 996) was used to record UV spectra of quercetin, gallic acid, benzoic acid, syringic acid, apigenin, tannic acid, caffeic acid, vanillin, rutin, malic acid, and ferulic acid. Through HPLC, plant samples were separated by using a column named Shim‐Pack CLC‐ODS (C‐18). The size of the column was 25 cm × 4.6 mm × 5 μm. Solvent A (H_2_O:acetic acid, 94:6, pH = 2.27), solvent B (acetonitrile 100%) were used as the mobile phase in chromatographic separation in which 15% solvent B (0–15 min), 45% solvent B (15–30 min), and 100% solvent B (35–45 min) were used with 1 ml/min flow rate. Then a UV–visible detector was used which has a wavelength (*λ*_max_) of 280 nm to separate phenolic compounds. Phenolic compounds were determined by comparing UV–visible spectra and the retention time of the peaks with UV–visible spectra and the retention time of standards. The quantification was performed by external calibration.

### 2.6 Minimum inhibitory concentration (MIC) of cladode, whole fruit, and pulp extracts against pathogenic bacteria

MIC was determined by the broth dilution method. This assay was performed using a 96‐well microtiter plate. Bacterial strains for MIC, that is, *Staphylococcus aureus* ATCC 6538, *Escherichia coli* ATCC 10536, *Salmonella typhi* ATCC 19430, and *Helicobacter pylori* NCTC 11637, were brought from the Institute of Microbiology, Faculty of Veterinary Science, University of Agriculture, Faisalabad, Pakistan.

For the assay, bacterial strains were cultured in Muller‐Hinton agar (MHA—pH 7.2) at 370°C. The stock culture slants were maintained at 4°C. The bacterial isolates were cultured in a Muller‐Hinton broth at 370°C for 4–6 h. The turbidity of the broth culture was adjusted to 0.5 (1–2 × 10^8^ CFU/mL) and used as standard inoculums for the antibacterial studies. One hundred microliters of broth were poured into wells of a microtiter plate. Sample dilutions (50, 25, 12.5, 6.25, 3.125, 1.56, 0.78, 0.39, 0.195, 0.09, and 0.05 μL) were prepared. From 1 to 11, columns were labeled with these dilutions. The sample was poured into columns from 1 to 11. Bacterial inoculum was prepared to the size of 10^4^–10^5^ CFU/mL. 5 μL of bacteria was poured into wells in columns 1–12. Plates were incubated at 37°C for 12–18 h. Growth was checked by naked eye evaluation.

### 2.7 Statistical analysis

All the experimental data were first tested for normality with Kolmogorov's test. Tukey's test was applied to calculate the significant differences in different parts of the plant. A general linear model (GLM) was performed to compare the effects of different extracts and parts of *Opuntia ficus indica* on phenolics, flavonoids, carotenoids, free radical scavenging activity, and MIC. The principal component analysis (PCA) and Pearson's correlation were conducted to evaluate the strength of the relationship between antioxidants and MIC of different parts of *Opuntia*. Data analysis was executed using statistical software Past 3 and Statistics 8.1. All measurements were recorded in triplicate (*n* = 3) and expressed as ±SD. The *p* < .01 was considered to be statistically significant.

## 3 RESULTS

### 3.1 Total phenolic content

**FIGURE 1:**
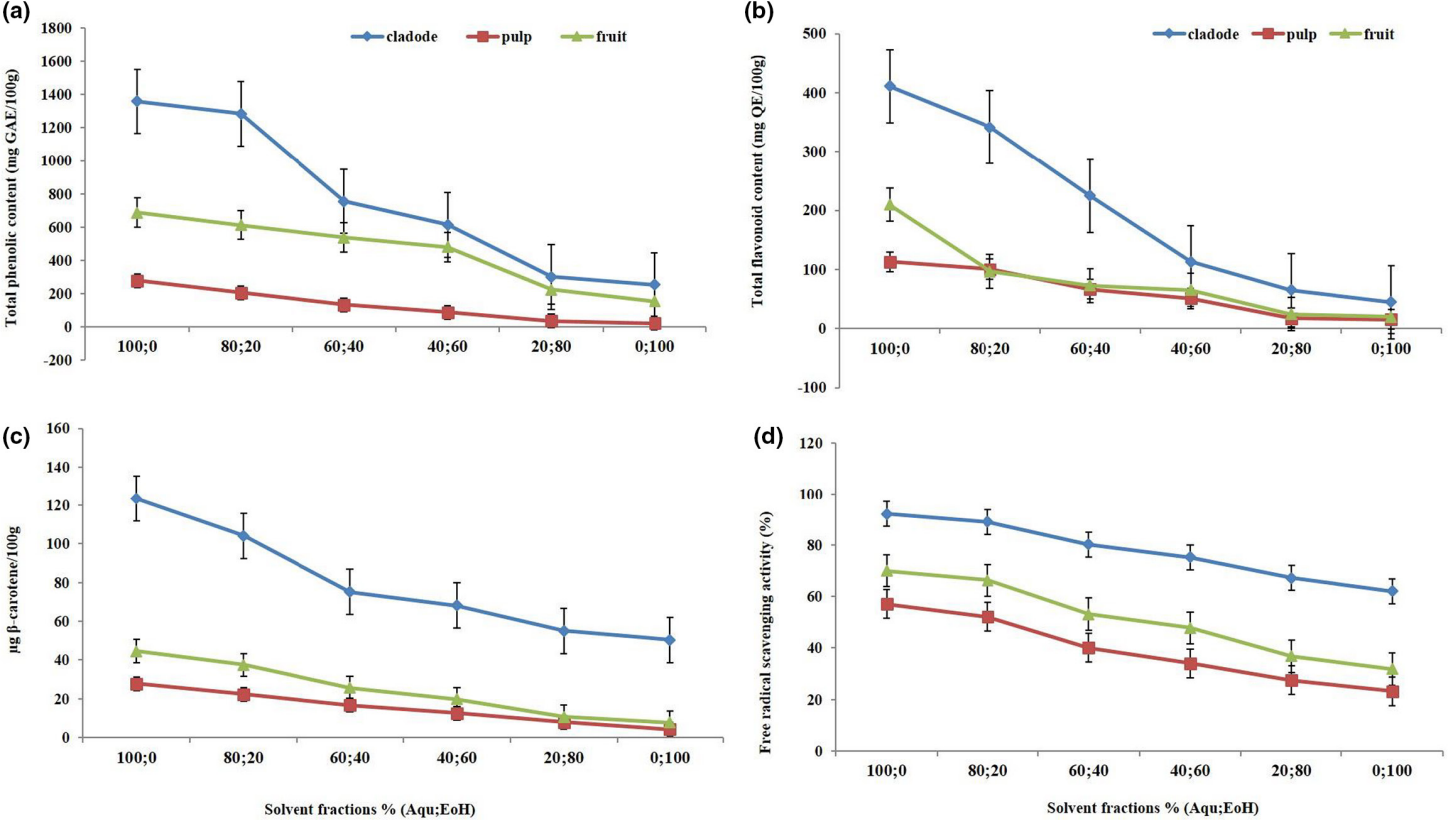
Determination of (a) total phenolic content, (b) total flavonoid content, (c) total carotenoids, and (d) free radical scavenging activity (DPPH) of hydroethanolic extracts of *Opuntia* cladode, pulp, and fruit

The results showed significant variation (*p* < .05) in the TPC of cactus pear's cladode, pulp, and fruit between parts and solvent fractions (Figure 1a; Table 1). Aqueous extracts yielded significantly the highest (*p* < .05) TPC values, whereas the lowest TPC value was exhibited by ethanolic extracts. A decreasing trend was observed among the solvent fractions with progressive change in ethanol concentration (*p* < .01). Regarding parts of cactus pear, it was observed that the statistically highest TPC value was found in the cladode part, whereas the lowest value was recorded in the case of pulp (*p* < .01).

### 3.2 Total flavonoid content

The results of TFC regarding the influence of parts of *Opuntia* cladode, pulp, and fruit are presented in Figure 1b and Table 1. It was evident from the results that TFC of cactus pear cladode, pulp, and fruit found to be significantly (*p* < .05) different among different parts of the prickly pear plant. The highest TFC was observed in cactus pear cladode, whereas the lowest value was observed in pulp (*p* < .01). With respect to solvent fractions, it was found that aqueous extracts yielded the highest TFC, whereas the lowest value was found in the case of ethanolic extracts (*p* < .01).

### 3.3 Total carotenoids content

*Opuntia* cladode contained significantly (*p* < .05) the highest carotenoids, whereas the pulp exhibited the lowest carotenoids (Figure 1c; Table 1). With respect to solvent fractions, it is also observed that the total carotenoid contents of cactus pear cladode, pulp, and whole fruit decreased significantly (*p* < .05) with a corresponding decrease in ethanol concentration (*p* < .01). Aqueous extracts exhibited the highest total carotenoid contents, whereas the lowest total carotenoid contents were found in ethanolic extracts.

### 3.4 Free radical scavenging activity (DPPH)

The results of the DPPH scavenging activity are presented in Figure 1d and Table 1. The highest DPPH activity was recorded in cactus pear cladode, while the lowest activity was observed in cactus pear pulp (Figure 1d and Table 1; *p* < .01). Among the solvent fractions, aqueous extracts showed the highest (*p* < .05) activity, whereas the lowest activity was observed in the case of ethanolic extracts (*p* < .01). A decreasing trend in DPPH free radical scavenging activity was observed with corresponding increases in the ethanol concentration within the solvent fractions.

### 3.5 Antimicrobial activity of the extracts

**FIGURE 2:**
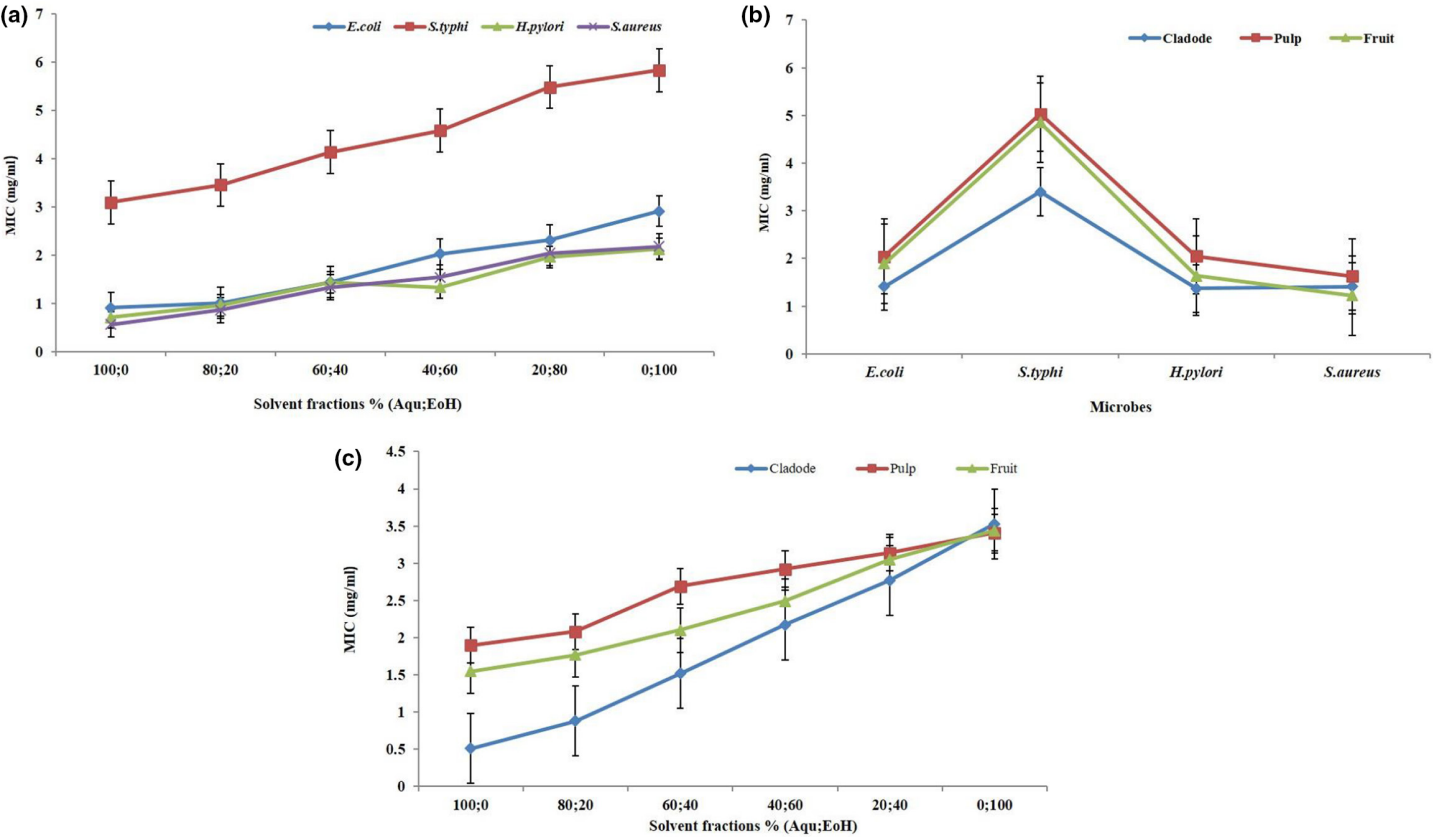
(a) Minimum inhibitory concentration (MIC) of hydroethanolic extracts of *Opuntia* cladode, pulp, and fruit (interaction between microbes and solvent fractions), (b) interaction between *Opuntia* parts and microbes, (c) interaction between *Opuntia* parts and solvent fractions

MIC of extracts was higher against *Staphylococcus aureus* followed by *Helicobacter pylori, Escherichia coli*, and *Salmonella typhi* (Figure 2a and Table 2). The extracts efficiency decreased significantly with a gradual increase in the ethanol concentration. A decreasing trend was observed among solvent fractions of cactus pear (*p* < .01 for solvent fractions).

**TABLE 2:** General linear model (GLM) evaluating the effect of different extracts and parts of *Opuntia ficus indica* on minimum inhibitory concentration (MIC) against different microorganisms

Source of variation	df	*F*	*p*
Microbes	3	288.84	.00
Parts	2	31.43	.00
Solvents	5	65.84	.00
Microbes × parts	6	6.92	.00
Microbes × solvents	15	1.17	.3010
Parts × solvents	10	3.02	.0017
Microbes × parts × solvents	30	0.91	.6049

### 3.6 Interaction between *Opuntia* parts and microbes

The results with respect to the interaction between microbes and cactus pear parts on MIC of hydroethanolic extracts of cactus pear cladode, pulp, and whole fruit are presented in Figure 2b and Table 2. Regarding microbes, statistically cactus pear extracts showed the highest MIC against *Staphylococcus aureus*, whereas the lowest MIC was observed in the case of *Salmonella typhi* (*p* < .01 for microbes). With respect to cactus pear parts, cladode exhibited higher MIC against different pathogens followed by pulp and whole fruit (*p* < .01) for cactus pear parts.

### 3.7 Interaction between *Opuntia* parts and solvent fractions

The results regarding the interaction between cactus pear parts and solvent fractions on the MIC of cactus pear cladode, pulp, and whole fruit are depicted in Figure 2c and Table 2. With respect to cactus pear parts, cladode showed the highest MIC against various pathogens, whereas the lowest MIC was found in pulp (*p* < .01 for cactus pear parts). Regarding solvent fractions, it was observed that aqueous extract exhibited maximum MIC against various pathogens, while ethanolic extract contained minimum MIC (*p* < .01 for solvent fractions). A declining trend in MIC against tested microbes was observed within the solvent fractions.

### 3.8 HPLC of phenolic compounds in prickly pear cladode, pulp, and whole fruit

The characterization and quantification of phenolic components of prickly pear cladode, pulp, and whole fruit was carried out using HPLC. The results showed that more phenolic compounds were observed in cactus pear cladode followed by pulp and whole fruit (Table 3). Dealing with the cladode extracts, these contained gallic acid, quercitin, benzoic acid, apigenin, caffeic acid, tannic acid, vanillin, and rutin as the major substances, while in the case of pulp, extracts contained gallic acid, quercitin, and rutin as major phenolic compounds. In addition, fruit extracts contained gallic acid, quercitin, syringic acid, and rutin as prominent phenolic compounds. These results demonstrated that there was great divergence among the phenolic compounds of *Opuntia* cladode, pulp, and fruit extracts. In the case of *Opuntia* cladode, 100% aqueous extract exhibited apigenin, caffeic acid, vanillin, tannic acid, quercitin, gallic acid, rutin, and benzoic acid in the highest amounts of 8.06, 6.01, 7.91, 6.67, 15.84, 125.58, 57.07, and 61.84 μg/g, respectively, while quercitin (2.78 μg/g), gallic acid (11.6 μg/g), and rutin (4.61 μg/g) were lower in pulp as presented in Table 3. Interaction of various solvent fractions and plant parts revealed that the aqueous extract of cladode showed the highest values of apigenin, caffeic acid, vanillin, tannic acid, benzoic acid, and rutin compared with the ethanolic extract of cladode which exhibited the lowest amounts of apigenin (0.36 μg/g), caffeic acid (0.62 μg/g), vanillin (1.03 μg/g), tannic acid (1.62 μg/g), quercitin (15.84 μg/g), gallic acid (66.19 μg/g), rutin (18.10 μg/g), and benzoic acid (18.51 μg/g). In the case of pulp, the aqueous extract exhibited the highest concentrations of quercitin (2.78 μg/g), followed by gallic acid (11.6 μg/g), rutin (4.61 μg/g) in comparison with ethanolic extracts of pulp which had the lowest amounts of quercitin (0.30 μg/g), gallic acid (1.67 μg/g), and rutin (0.58 μg/g). In the case of whole fruit, rutin, quercitin, gallic acid, and syringic acid were detected in aqueous as well as ethanolic extracts. Regarding prickly pear whole fruit, the aqueous extract exhibited the highest amounts of rutin (7.43 μg/g), quercitin (3.41 μg/g), gallic acid (84.74 μg/g), syringic acid (23.54 μg/g), while ethanolic extract showed the low quantity of rutin (0.40 μg/g), quercitin (0.19 μg/g), gallic acid (29.22 μg/g), syringic acid (5.18 μg/g).

**TABLE 3:** HPLC analysis of phenolic compounds of cactus pear cladode, pulp, and whole fruit

Solvent fractions (%)	Cladode							
	Apigenin	Caffeic acid	Vanillin	Tannic acid	Quercitin	Gallic acid	Rutin	Benzoic acid
Aqueous (100)	9.51 ± 0.43a	7.15 ± 0.12a	7.07 ± 0.97a	6.69 ± 0.92a	17.57 ± 0.64a	122.47 ± 2.65a	55.89 ± 1.99a	64.35 ± 4.14a
80	8.80 ± 0.32a	5.97 ± 0.31ab	6.89 ± 0.26ab	6.19 ± 0.55ab	12.89 ± 0.18b	120.23 ± 2.35ab	54.00 ± 1.66a	57.34 ± 3.02b
60	5.65 ± 0.33ab	5.57 ± 0.42abc	5.79 ± 0.32ab	5.72 ± 0.47ab	12.61 ± 0.29b	118.96 ± 1.36ab	53.93 ± 0.78a	56.74 ± 3.04b
40	4.28 ± 0.42b	3.57 ± 0.22b	5.64 ± 0.24ab	5.29 ± 0.30ab	11.07 ± 0.34b	115.53 ± 1.04b	50.71 ± 0.59b	53.90 ± 2.89c
20	3.00 ± 0.20b	3.09 ± 0.43bc	3.09 ± 0.23b	2.80 ± 0.30c	6.34 ± 0.23c	83.97 ± 0.66c	24.27 ± 0.45c	33.97 ± 2.23d
Ethanol (100)	2.41 ± 0.15c	1.79 ± 0.20d	3.07 ± 0.09b	2.62 ± 0.26c	5.43 ± 0.10c	68.89 ± 0.53d	17.78 ± 0.40d	20.05 ± 1.32 e

Solvent fractions (%)	Pulp							
	Quercitin	Gallic acid	Rutin					
Aqueous (100)	5.52 ± 0.49a	11.6 ± 0.37a	6.95 ± 0.21a	—	—	—	—	—
80	4.69 ± 0.30a	7.77 ± 0.61b	6.20 ± 0.34a	—	—	—	—	—
60	4.50 ± 0.33a	5.82 ± 0.49b	6.16 ± 0.22ab	—	—	—	—	—
40	4.13 ± 0.30a	3.90 ± 0.58c	5.60 ± 0.20ab	—	—	—	—	—
20	2.88 ± 0.28b	2.25 ± 0.53bc	2.68 ± 0.03c	—	—	—	—	—
Ethanol (100)	2.16 ± 0.30b	1.67 ± 0.44d	2.15 ± 0.01c	—	—	—	—	—

Solvent fractions (%)	Whole fruit							
	Rutin	Quercitin	Gallic acid	Syringic acid				
Aqueous (100)	8.40 ± 0.45a	7.21 ± 0.45a	88.21 ± 3.97a	26.65 ± 0.52a	—	—	—	—
80	5.40 ± 0.41b	5.13 ± 0.35b	84.24 ± 3.26a	23.27 ± 0.35b	—	—	—	—
60	4.40 ± 0.32bc	4.53 ± 0.36b	78.19 ± 3.32b	18.82 ± 0.32bc	—	—	—	—
40	3.04 ± 0.23c	3.73 ± 0.54ab	73.30 ± 3.24bc	15.80 ± 0.15d	—	—	—	—
20	1.12 ± 0.27d	1.34 ± 0.26c	68.73 ± 2.23c	9.30 ± 0.21 e	—	—	—	—
Ethanol (100)	1.03 ± 0.07d	1.19 ± 0.23c	32.57 ± 0.52d	7.13 ± 0.33f	—	—	—	—

*Note*: Mean values are the result of three replications (*n* = 3) and shown as mean ± SD. Means carrying the same small letters (a–f) in the column are significantly (*p* < .05) different from each other.

### 3.9 Principle component analysis

**FIGURE 3:**
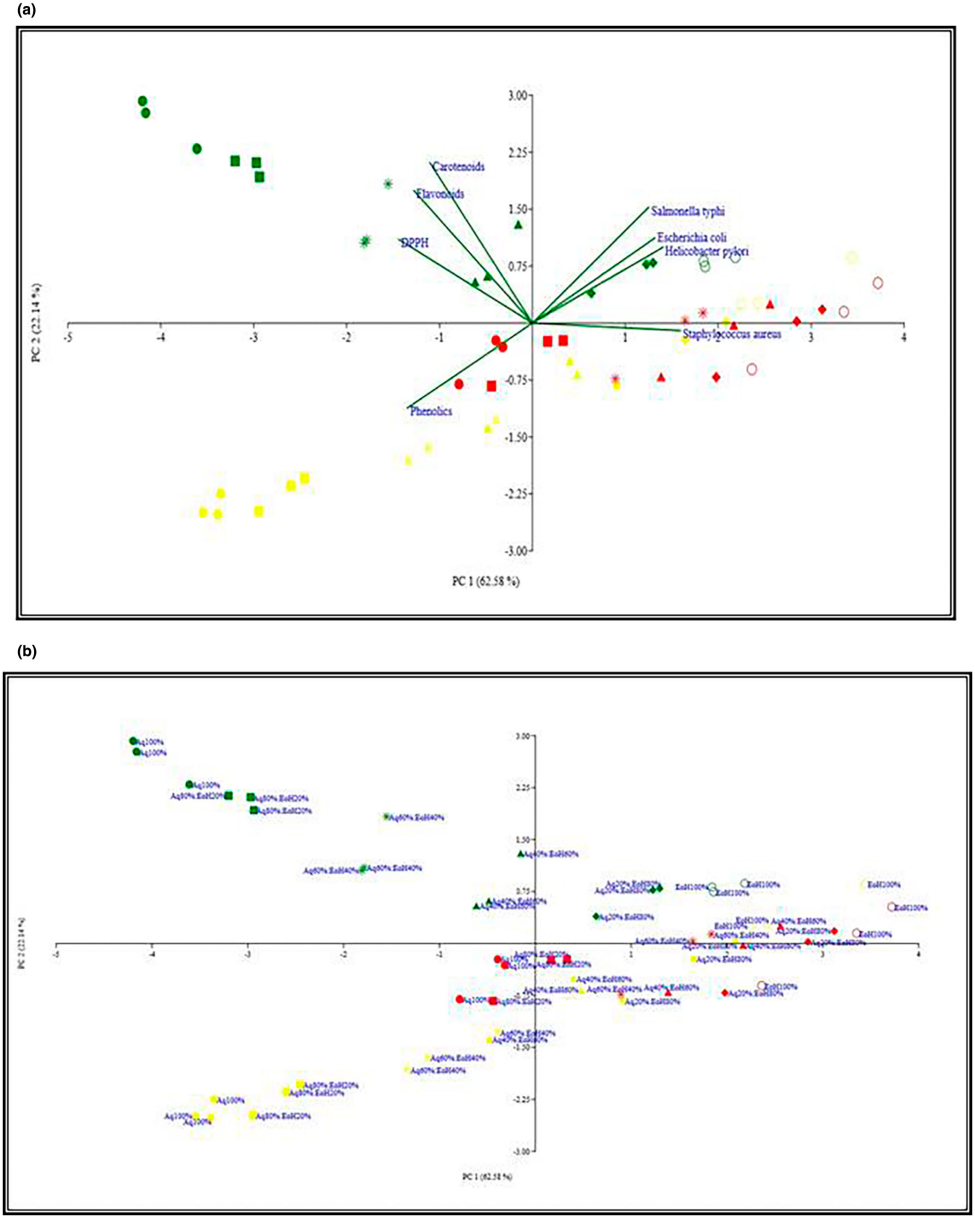
Principle component analysis (PCA) bi‐plot shows the relationship between antioxidant activity and microbes (a), MIC, and aqueous and ethanolic extracts of *Opuntia ficus indica* parts (b).

Principle component analysis (PCA) was used to determine the correlation between antioxidant activity, MIC, and the various hydroethanolic extracts. PCA bi‐plot showing the relationship between antioxidant activity and microbes is presented in Figure 3. PC 1 (62.58%) and PC 2 (22.24%) explained 84.82% of the total variation (Figure 3a,b). The PCA results showed a clear separation between different microbes namely, *Salmonella typhi, Escherichia coli, Helicobacter pylori*, and *Staphylococcus aureus* and antioxidants, that is, carotenoids, flavonoids, DPPH, and phenolic compounds along the PC1. *Salmonella typhi, Escherichia coli*, and *Helicobacter pylori* showed almost similar responses. The variation in PC2 was associated with *Staphylococcus aureus* response. The results indicated that most of the solvent fractions of fruit are located far from the flavonoids, carotenoids, phenolics, and DPPH, whereas solvent fractions of cladode and fruit are closely clustered to microbes. Aqu 100%, Aqu 80%; EoH 20%, Aqu 60%; EoH 40%, and Aqu 40%; EoH 60% of fruit showed a negative correlation with that of cladode along the PC1 axis, while on this axis, Aqu 20%; EoH 80%, EoH 100% of fruit, Aqu 40%; EoH 60%, Aqu 20%; EoH 80%, EoH 100% of pulp were strongly correlated with each other showed a positive correlation with that of cladode. Aqu 40% and EoH 60% of fruit showed a negative correlation with that of pulp compared with cladode. Aqu 20%; EoH 80% and EoH 100% of fruit, Aqu 20%; EoH 80%, Aqu 60%; EoH 40% and EoH 100% of pulp and Aqu 20%; EoH 80%, Aqu 40%; EoH 60% and Aqu 60%; EoH 40% of cladode were positively correlated with each other showed a positive correlation with that of pulp.

## 4 DISCUSSION

Total phenolic contents were measured in gallic acid equivalent due to their acidic stability and good response to the Folin–Ciocalteu reagent. The quantity of TPC found in the aqueous extract of cactus pear cladode was higher followed by cactus pear whole fruit and pulp which might be attributed to the presence of mucilage content in cladode. Mucilage is directly related to the moisture content because heteropolysaccharide components of cactus pear cladode have the ability to absorb water (Mariel et al., 2017). The present results regarding cactus pear pulp are consistent with those obtained by Lamia et al. (2018) who observed that the methanolic pulp extract of *Opuntia ficus indica* contained about 54.33 ± 2.51 mg GAE/100 g, whereas *Opuntia streptacantha* exhibited TPC of about 104.66 ± 1.52 mg GAE/100 g DW. Teresita et al. (2010) observed that the Blanco and Manso commercial varieties of cladode had total phenolic contents of 5.25 and 11.7 mg GAE/g DW, respectively, that follow the same pattern as observed in the current study. TPC is considered to play a defensive role against tissue damage (Asif et al., 2017). Much higher values were obtained in cladode when Du Toit et al. (2018) determined total phenolic content in different varieties of *Opuntia* cladode. The differences among the TPC of three parts of *Opuntia* might be attributed to the variability in solvent types and polarity employed, plant varietal differences, and respective extraction methods utilized for the extraction of polyphenolic compounds. It might be implied from the aforementioned findings that a pivotal role was played by the solvent polarity for maximizing the solubility of polyphenols and binary phases of employed organic solutions proved to be more effective in getting better extraction yields of phenolic‐rich extracts. In addition, it was also evident that *Opuntia* cladode showed the most promising and remarkable potential with respect to polyphenolic compounds compared with pulp and whole fruit and this suggested that *Opuntia* cladode could be a rich source of polyphenolic compounds regardless of the type of solvent employed for extraction purpose (El‐Beltagi et al., 2019).

Flavonoids are well‐recognized active components of plants that have played a medicinal role in human bodies. Cladode possessed more total flavonoid content which might be due to more metabolism in this part of the plant resulting in high metabolite production. Although total flavonoid contents in cladode are less than total phenolic contents, even a small concentration of total flavonoid contents possesses strong antioxidant potential (Asif et al., 2017; Dhaouadi et al., 2013). TFC of the aqueous extract of cactus pear cladode in the present study was slightly lower (410.71 ± 50.78 mg QE/100 g DW) than that observed by Ben Lataief et al. (2021) (6.45 ± 0.23 mg CE/g DW). The results regarding TFC of whole fruit and pulp are in good agreement with that reported by Elazzouzi et al. (2002) and Lamia et al. (2018) who observed that TFC detected in *Opuntia* pulp was 22.47 ± 2.1 mg RE/100 g DW. These variations in TFC might be due to the solubility capacity of matrix components and the polarity of a hydroethanolic mixture (Luque‐Rodriguez et al., 2007; Nur‐Izzati et al., 2021; Shi et al., 2003; Toure et al., 2015). Previously, reported findings have also suggested that solvent polarity maximizes the extraction yields of flavonoids. The earlier scientists also observed that variation in quantities of TFC might be connected to the differences in extraction methods, climatic factors, and soil chemistry of the regions (Chavez‐Santoscoy et al., 2009; Haile et al., 2016; de Wit et al., 2019; Guevara‐Figueroa et al., 2010; Kuti, 2004).

The results of the present study regarding total carotenoids of cladode are in line with the previous studies conducted by Barba et al. (2022) who found that the cladode of all cultivars contained more carotenoids than fruit pulp and peel. A similar trend was observed by Medina‐Torres et al. (2011) who compared cladode to other vegetables and it was demonstrated that cladode had higher carotenoids than baby beetroot, carrots lettuce, and spinach. The cladodes had several times more carotenoids compared with that fruit pulp. The discrepancies in these reported carotenoid levels might be due to differences in the ripeness of the fruit because the carotenoid levels can change dramatically during ripening (Mabrouki et al., 2015; Lanuzza et al., 2017; Vuong et al., 2002). The degree of ripeness is thought to be a factor influencing the discrepancies in carotenoid quantity in *Opuntia*. This is consistent with the findings of Rodriguez et al. (1976) who studied carotenoids in bitter melon and reported that the number of carotenoids increased from five in the immature cladode to six at the mature‐green and to 14 at the partly ripe to ripe stages (Vuong et al., 2002).

**FIGURE 4:**
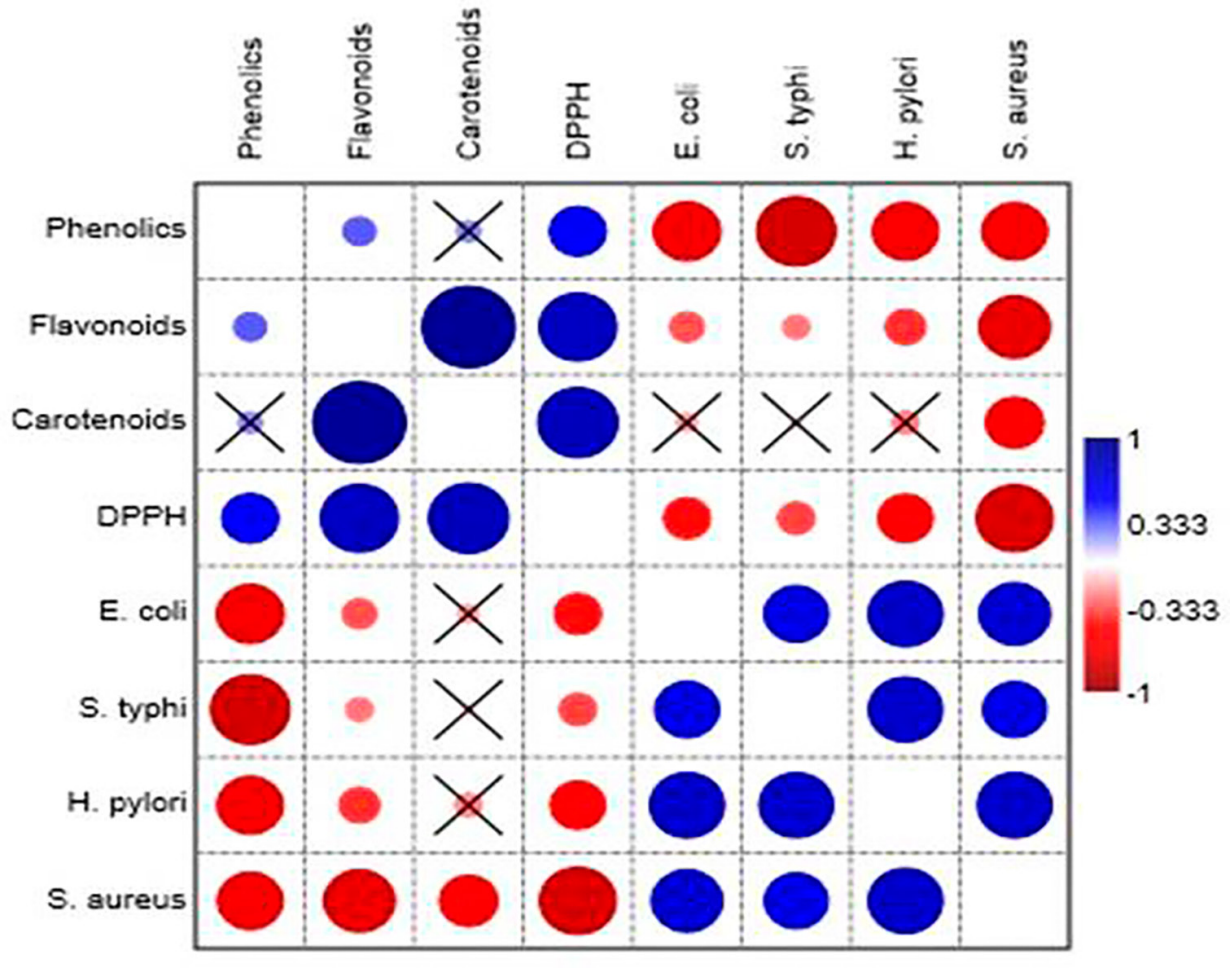
Pearson's correlation coefficient analysis among antioxidant activity and minimum inhibitory concentration (MIC) responses in *Opuntia ficus indica* parts

Naturalistic antioxidants like phenolics, flavonoids, and carotenoids are found in different plant products (Farag et al., 1989; Jeong et al., 2004) and these are widely reported to preserve components of food which are able to oxidize easily because of oxidation. This effect differs vastly relying on the growing conditions, extraction process, and a multitude sides of the chemical structure of the active constituents, that is, the amount and position of hydroxyl groups, molecular weight, particle size, solvent concentration, time of contact, temperature, and mass‐solvent ratio (Soto‐García & Rosales‐Castro, 2016; Frankel & Meyer, 2000; Madhavi & Salunkhe, 1995). In the present work, *Opuntia* cladode, pulp, and fruit hydroethanolic extracts as a source of natural antioxidants were evaluated. Considering the data in Figure 4, cactus pear cladode exhibited the highest antioxidant activity assessed by the aforementioned method in comparison with whole fruit and pulp which might be attributed to the presence of high content of flavonoid in cladode as observed in the current study. Some other researchers reported that flavonoids (quercitin) are the main compounds responsible for the scavenging activity of cladode (Ghosal & Mandal, 2012; Azizah et al., 1999; Lee et al., 2002). It is also supported by the findings of earlier research by Jaramillo‐Flores et al. (2003) that processed cladodes are a good source of antioxidants that might be due to the fact that mucilage present in cladode captures antioxidants and increased the free radical scavenging activity of cladode as seen in the current research. The same trend was seen by earlier scientists (Du Toit et al., 2018; Haile et al., 2016) who stated that the cladodes samples have good antioxidant activity, ranging from 59.3% to 85.8% inhibition compared with ascorbic acid. The findings of the present study regarding the radical scavenging activity of cactus pear pulp are well supported by Koshak et al. (2020).

It appears from the results of the present study that there is a correlation between the antioxidant capacity and the chemical structures of phenolic compounds. The evidence for this compositional requirement is supported by Bendary et al. (2013) who mentioned that phenolic components are excellent electron donors due to the ability of their hydroxyl groups to participate in antioxidant processes. In this context, Benjakul et al. (2014) reported that the differences in chemical structures and numeral of the hydroxyl groups in phenolic components contribute to the diversity in their antioxidant activity. It is worth mentioning that Derakhshan et al. (2018) mentioned that there is a significant positive relation between antioxidant ability and total phenols and that confirms the findings of the present study which indicated that *Opuntia* cladode contained high quantities of total phenols and possessed the highest antioxidant activity in comparison with other plant parts under study.

In the present study, a significant quantity of polyphenols was detected in the cladode part of cactus pear in aqueous ethanolic fractions. It is worth mentioning that the HPLC results under study agreed quite well with the findings of Francesco et al. (2020) who illustrated that *Opuntia ficus indica* contained a high quantity of polyphenols. The present study results are consistent with those of Nadia et al. (2019). Larbi et al. (2017) mentioned that 70% ethanolic extract of cladode contained quercitin, gallic acid, tannic acid, apigenin, caffeic acid, and vanillin. The findings of the current research are in close association with the work of previous researchers (Larbi et al., 2017) who concluded that *Opuntia* cladodes are a rich source of bioactive components which can play an important part in the human diet. Additionally, the variation observed in polyphenols among different fractions is affected by extraction time and temperature as reported by Robards (2003).

Phytochemical screening revealed that several classes of secondary metabolites exist, such as flavonoids, phenolics, and carotenoids. Several molecules are active on pathogenic microorganisms (Awouafack et al., 2013; Erfan & Marouf, 2019; Tsopmo et al., 2013; Qayyum et al., 2016; Syukriah et al., 2014). The presence of these phytochemicals in the tested plant extracts can give a preliminary explanation of their antimicrobial activities. Differences were observed in the antibacterial activities of the extracts. These could be due to the differences in their chemical composition as well as in the mechanism of action of their bioactive constituents. All the extracts are rich in different phytochemicals; however, activity does not depend only on these phytochemicals in the plant extracts but also on their concentration and the possible interaction with other components (Dangoggo et al., 2012; Rodriguez‐Amaya et al., 2001; Simoes et al., 2009). In this study, the antimicrobial findings of *Opuntia ficus indica* extracts at different concentrations have the ability to inhibit the growth of *Salmonella typhi, Helicobacter pylori, Staphylococcus aureus*, and *Escherichia coli*. Against *Staphylococcus aureus*, the highest antibacterial activity of the hydroethanolic extracts of cactus pear cladode, pulp, and fruit was recorded, whereas the lowest activity was seen against *Salmonella typhi*. The antibacterial results showed that the cladode of *Opuntia ficus indica* showed the least MIC value, whereas pulp and whole fruit showed higher MIC values against *Salmonella typhi, Helicobacter pylori, Escherichia coli*, and *Staphylococcus aureus*. The lower the MIC, the higher the efficiency against pathogens. Variation in MIC values against tested pathogenic strains in the present study might be due to variation extraction methods, size of inoculum, incubation length, and range of solvent quantity used as reported by Moosazadeh et al. (2014) and Kalil et al. (2014). Low antimicrobial action of extracts might be due to the variation in the type of solvent used, unreleased/bound phenols in extract matrices (Avila‐Nava et al., 2014; Zeghad et al., 2019), and/or inability of extracted compounds to diffuse into the antibacterial assay medium (Kurek et al., 2011). Aqueous extracts exhibited significantly (*p* < .05) higher efficiency followed by ethanolic extracts against all tested pathogenic bacteria as indicated in Figure 4a. The aqueous extract activity showed that the solubility of antimicrobial potential components present in *Opuntia* is high in water. Mukonowenzou et al. (2021) proposed that the capacity of water to extract different antimicrobial components reported in the current research is well supported by earlier research concluding that water is a good solvent to extract antimicrobial components from medicinal plants (Mukonowenzou et al., 2021; Yu et al., 2014). The findings also revealed that *Staphylococcus aureus* showed more sensitivity to cactus pear fruit and pulp followed by *Helicobacter pylori, Escherichia coli, and Salmonella typhi*, whereas *Helicobacter pylori* showed more sensitivity to cladode. *Staphylococcus aureus* was easily inhibited at the lowest concentration in the case of fruit, whereas in the case of pulp the highest concentration of the extract was required to inhibit growth. On the other hand, a high concentration of extracts was required to inhibit the growth of *Salmonella typhi*. This might be due to differences in their sensitivity to antibacterial agents. The results of this study are in line with the findings of Karima et al. (2013). A similar trend was observed by Taguri et al. (2004) who reported that Gram (+) was more sensitive than Gram (−) bacteria. *Staphylococcus aureus* as Gram (+) bacteria is famous due to its high sensitivity to phenolic extracts. Generally, Gram (−) is more resistant to bactericidal polyphenols than Gram (+) bacteria. According to Taguri et al. (2004), the average MIC values of extracts indicated that *Staphylococcus aureus* (192 μg/ml) are more susceptible to polyphenols followed by *Salmonella typhi* (795 μg/ml) and *Escherichia coli* (1519 μg/ml). The present findings are in conformity with the results of Taguri et al. (2004). Ikigai et al. (1993) proposed that two factors, such as repulsion between lipopolysaccharide‐coated surfaces of Gram (−) bacteria and the phenolics are responsible for this difference. It was reported in another report that gram‐positive bacteria have a cell wall made up of peptidoglycan which helps in cell wall penetration (Koubaa et al., 2015). The phenolic composition of plant extracts might be responsible for their inhibitory effect against pathogenic bacteria (Rodriguez et al., 1976). The inhibitory effect of these phenolics could be explained by adsorption to cell membranes, interaction with enzymes, or deprivation of substrate and metal ions (Baydar et al., 2004).

To obtain an overall perspective of the free radical capacity and the respective chemical constituents, pairwise correlations among total phenolic content, total flavonoids content, total carotenoids, antioxidant activity, and MIC were performed. The results of the present study revealed that flavonoids, carotenoids, and DPPH were clustered together but did not seem to be associated with phenolics. On the other hand, *Salmonella typhi, Helicobacter pylori, Escherichia coli*, and *Staphylococcus aureus* are clustered together. The interesting property of the extracts is that they have low MIC and possessed weak correlation and a lower value of MIC indicated that these extracts have high antibacterial activity against microbes. All microbes had a significant negative correlation with phenolics. These results are in concordance with the previous study conducted by Daglia (2012) who reported that phenolics have been shown to possess strong antibacterial activity. Dutta, Ghosal, Mitali & Palash (2012) also found a high correlation between total phenolics and DPPH free radical scavenging activity suggesting that phenolic compounds are the major contributors to antioxidant activity. Another study conducted by Gil et al. (2002) reported that phenolics had a significant correlation with DPPH. The correlation of the DPPH assay with total phenolics and total flavonoids was positive, demonstrating that this assay can be considered for measuring the free radical scavenging capacity of *Opuntia*. It is also noteworthy that Aqu 100% of fruit are located far on the left of the figure meaning that Aqu 100% had a weak association with all antioxidants and MIC results. It was also observed that all solvent fractions seem to cluster together except solvent fractions of the fruit. On the other hand, most of the solvent fractions of *Opuntia* pulp are located far on the right of the figure meaning that pulp had weak relation with microbes but solvent fractions of cladode and fruit are closely associated with microbes. Polyphenols determined in te present study have a strong correlation with the antioxidant activity of *Opuntia*. These results are in agreement with previous studies that reported that phenolic compounds played a crucial role in scavenging free radicals (Bouabid et al., 2018). These results might be ascribed to the different levels of concentration (Du Toit et al., 2018).

## 5 CONCLUSION

The present work was focused on evaluating the resultant extracts obtained from the extraction of prickly pear (*Opuntia* cladode) pulp and fruit as a source of natural antioxidants. In general, HPLC data showed that prickly pear contained a high quantity of bioactive compounds. *Opuntia* cladode contained high amounts of total phenols, total flavonoids, and total carotenoids and also exhibited strong antioxidant potential. Regarding prickly pear whole fruit, the aqueous extract exhibited the highest amounts of rutin (7.43 μg/g), quercitin (3.41 μg/g), gallic acid (84.74 μg/g), syringic acid (23.54 μg/g), whereas ethanolic extract showed the low quantity of rutin (0.40 μg/g), quercitin (0.19 μg/g), gallic acid (29.22 μg/g), and syringic acid (5.18 μg/g). Moreover, prickly pear possesses effective antibacterial bioactive constituents against multi‐drug‐resistant bacteria and can be used for the prevention of different infectious diseases. The correlation analysis demonstrated phenolic contents, flavonoid contents, and carotenoids exhibited a substantial positive relationship with DPPH in an increasing manner. In this context, cladode is a valuable source of health‐promoting compounds, fulfilling concurrently the promising antioxidant activity that can be utilized virtually as food complements, to tardiness lipid oxidization and healing from particular ailments via its free radicals scavenging ability. It would be interesting to conduct more research to inspect the role of bioactive components which responsible for these activities. Hence, more studies are necessary to estimate the antioxidant and antimicrobial efficiencies of their individual purified fractions. Furthermore, it was revealed in the current study that the concentration of phenolic compounds and antioxidant activity in prickly pear extracts is sufficient to be considered a possible source of antioxidant supplements. In general, it may have the potential to contribute to better bioactive compounds contents in the diet of children, mothers, and adolescents to combat mineral deficiency problems and prevent many diseases including osteoporosis and cardiovascular disorders. The demand for fortified foods in the market is huge. Hence, prickly pear extracts fortification in foodstuffs is a convenient way to eradicate malnutrition. This abundant tree in Pakistan can become a great source of income for the nation if this potential for highly nutritional food is exploited by industries and researchers.

## FUNDING INFORMATION

There was no funding received for this study from any funding organization.

## CONFLICT OF INTEREST

The authors declare that they have no conflict of interest to disclose.

## Data Availability

The data used to support the findings of this study are available from the corresponding author upon reasonable request.
